# Sensing a Changing World

**DOI:** 10.3390/s90906819

**Published:** 2009-08-28

**Authors:** Arend Ligtenberg, Lammert Kooistra

**Affiliations:** Centre for Geo-Information, Wageningen University, Droevendaalsesteeg 3, 6708 PB Wageningen, The Netherlands; E-Mail: lammert.kooistra@wur.nl

The workshop “Sensing a Changing World” was held in Wageningen, The Netherlands, from November 19–21, 2008. The main goal of the workshop was to explore and discuss recent developments in sensors and (wireless) sensor networks for monitoring environmental processes and human spatial behavior in a changing world. The challenge is then to develop concepts and applications that can provide timely and on-demand knowledge to end-users in different domains over a range of different spatial and temporal scales.

During this workshop over 50 participants, representing 15 countries, presented and discussed their recent research. The workshop provided a broad overview of state-of-the-art research in a broad range of application fields: oceanography, air quality, biodiversity and vegetation, health, tourism, water management, and agriculture. In addition the workshop identified the future research challenges.

One of the outcomes of the workshop was a special issue in the journal *Sensors* with contributions presented at the workshop. This editorial of the special issue aims to provide an overview of the discussions held during the workshop. It highlights the ideas of the authors and participants of the workshop about directions of future research for further development of sensor-webs for “sensing” spatial phenomena. The “big” question was are we already able to sense a changing world? And if the answer is positive, then what are we going to sense and for what?

## Current progress

**Figure 1.**
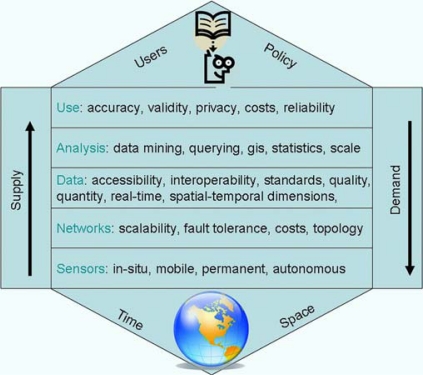
A multi level view of a sensor web.

Before answering the above noted questions, it is good to reflect on what we have encountered and accomplished already. Therefore we might look at sensor webs for environmental monitoring as a multi leveled system (Figure 1). The general aim of a sensor web is to inform users about the condition of the spatial-temporal aspects of the physical environment. At the bottom of Figure 1 physical measurement is done by either an *in-situ* or a mobile sensor [1]. At this level research is mainly conducted by physical sensor research aiming at creating accurate, robust, energy-efficient and reliable measuring devices. For the domain of environmental sciences the work done at this level needs to be taken for granted. The second layer deals with the design and implementation of sensor webs. To cover a certain geographical extent or multifaceted domain, sensors might be coupled into a sensor network. Sensors in such a network should be able to exchange information efficiently and effectively. Research here focusses on the design of scalable, fault tolerant and cost efficient network topologies consisting of sets of static or mobile sensors [2]. The third layer offers the concepts, methods, and tools to enable access to data. Research here is concerned with methods to make accessible the often large quantities of sensor data through unified or interoperable interfaces. Databases optimized to handle often real-time spatial temporal data is current important fields of research. At the fourth level research is concerned with analytical techniques and tools to generated knowledge from the often large amounts of sensor data. Novel approaches like data mining, specialized query languages, etc. need to be further developed. Especially the connection with geographical information systems (GIS) needs attention. Additional analytical, cartographic visualization techniques are necessary to analyze and visualize the processed sensor data. The final level concerns the aspects related to the direct use of the data and the results of the analyses by the end users. Privacy needs to be ensured for data about human activities and, depending on the application, certain levels or reliability and accuracy need to be achieved to be useful.

During the workshop the following general issues were raised concerning the current status of sensor web research:

- The definition of sensors and sensor webs is very broad;
- it covers a very broad area of research;
- involving many scales and extremes;
- involving many domains;
- and is currently mainly technology driven.

Many of these issues are reflected in the 11 papers of this special issue and the proceedings of the workshop. Sensors and sensor webs are encountered in a broad range of applications varying from specialized *in-situ* sensors capable of collecting specific point based measurements [3], to mobile sensors capable of collecting data about spatial-temporal parameters [4–6], to remote sensing sensors mounted on airplanes and satellites able to collect multifaceted information about large geographical areas at the same time [7]. At the same time, however, many of the current sensor webs are defined mainly to function as a dedicated and stand alone network. The need for better integration of sensors and sensor webs is recognized. Concepts and techniques on metadata [2], data structures and languages [8] are raised. Also discovery mechanisms [9] and the connection with geo-data infrastructures are recognized as being important.

## Research directions

Research about sensors and sensor webs might be categorized into three rather broadly defined areas of application. The first area aims at the development of sensor webs in order to better measure known phenomena. The question of how to improve sensors networks in terms of their accuracy, efficiency and intelligence is of importance here. In a technological sense, energy consumption and the robustness of sensor networks is still pending issues. It is not only a matter of better electronics and batteries but also of more robust, self healing, and fault tolerant network topologies and notification strategies.

Secondly, there are the ideas of sensors as a “sensing skin” and an “environmental microscope” on the Earth. Integration of dynamic and heterogeneous sensor webs measuring various aspects, at different scales, and within different domains are key aspects. Pending issues at the technical levels are the development of ontologies for the interoperability of data and integration of and reasoning about knowledge, and the implementation of standardized, easily accessible sensor webs. Current standards like the Sensor Web Enablement (SWE) architecture, the Sensor Model Language (SensorML), or the Sensor Observation Services (SOS) of the Open Geospatial Consortium (OGC) mainly focus on the syntactical aspect of sensor webs. The questions “what do we want to know”, “when do we want to know” and “how do we want to know” are largely unsolved and can only be answered by the applied domains. Also the concept of humans as sensors and informal sensor networks are interesting in this context.

The third aspect is the use of sensor webs to discover new phenomena about the world. For example the use of mobile phones and GPS tracking to analyze the behavior of people roaming around in an area might yield additional insight beyond the possibilities of current data and analysis techniques. Data mining techniques, visual analytics techniques and additional knowledge discovery techniques need to be further developed to make this potential new source of information applicable for monitoring and analysis of human-environment relations. Important questions include how privacy can be guaranteed when using movement information and what will society accept.

We would like to conclude that all participants of the workshop recognized the development of sensor webs as an important potential valuable addition to the existing toolbox for the environmental sciences; and that adding the dimension of (near) real-time spatial-temporal information about the environment, gives the opportunity to improve the monitoring of critical environmental processes. However sensor web technology is for the most part still in its infancy especially regarding the interoperability and semantic aspects of sensor webs.

## References

[1] Honda K., Shrestha A., Witayangkurn A., Chinnachodteeranun R., Shimamura H. (2009). Fieldservers and Sensor Service Grid as Real-time Monitoring Infrastructure for Ubiquitous Sensor Networks. Sensors.

[2] Ballari D., Wachowicz M., Callejo M.A.M. (2009). Metadata behind the Interoperability of Wireless Sensor Networks. Sensors.

[3] Kotamäki N., Thessler S., Koskiaho J., Hannukkala A., Huitu H., Huttula T., Havento J., Järvenpää M. (2009). Wireless in-situ Sensor Network for Agriculture and Water Monitoring on a River Basin Scale in Southern Finland: Evaluation from a Data User's Perspective. Sensors.

[4] Lammeren van R., Goossen M., Roncken P., Kooistra L., Ligtenberg A. (2008). Enhancing the Landscape Experience by Location Based Services. International Workshop Sensing a Changing World 2008.

[5] Ligtenberg A., Kooistra L., Ligtenberg A. (2008). The Use GPS for the Analyses of Movements of Visitor Flows in Nature Areas. International Workshop Sensing a Changing World 2008.

[6] van der Spek S., van Schaick J., de Bois P., de Haan R. (2009). Sensing Human Activity: GPS Tracking. Sensors.

[7] Kooistra L., Bergsma A., Chuma B., de Bruin S. (2009). Development of a Dynamic Web Mapping Service for Vegetation Productivity Using Earth Observation and in situ Sensors in a Sensor Web Based Approach. Sensors.

[8] Baumann P. (2009). Language-Based Access to Large Sensor Repositories. Sensors.

[9] Jirka S., Bröring A., Stasch C. (2009). Discovery Mechanisms for the Sensor Web. Sensors.

